# The roles of the dietitian in an 18-week telephone and mobile application nutrition intervention for upper gastrointestinal cancer: a qualitative analysis

**DOI:** 10.1007/s00520-023-07684-9

**Published:** 2023-03-29

**Authors:** Sharni Testa, Kate Furness, Tammie Choi, Terry Haines, Catherine E. Huggins

**Affiliations:** 1 Department of Nutrition, Dietetics and Food, Monash University, Clayton, VIC Australia; 2 Nutrition and Dietetics, Monash Medical Centre, Monash Health, Clayton, VIC Australia; 3 Department of Physiotherapy, School of Primary and Allied Health Care, Faculty of Medicine, Nursing and Health Sciences, Monash University, Frankston, VIC 3199 Australia; 4 Department of Nursing and Allied Health, School of Health Sciences, Swinburne University of Technology, Hawthorn, VIC Australia; 5 School of Primary and Allied Health Care, National Centre for Healthy Ageing, Monash University, Level 3 Building G, McMahons Road, Frankston, VIC 3199 Australia; 6 School of Health and Social Development, Faculty of Health, Institute for Health Transformation, Deakin University, Melbourne, VIC Australia

**Keywords:** Upper gastrointestinal cancer, Nutritional support, Telehealth, Qualitative research, Professional patient relationship

## Abstract

**Purpose:**

This study aimed to explore the patient-dietitian experience during an 18-week nutrition counselling intervention delivered using the telephone and a mobile application to people newly diagnosed with upper gastrointestinal (UGI) cancer to (1) elucidate the roles of the dietitian during intervention delivery and (2) explore unmet needs impacting nutritional intake.

**Methods:**

Qualitative case study methodology was followed, whereby the case was the 18-week nutrition counselling intervention. Dietary counselling conversations and post-intervention interviews were inductively coded from six case participants which included fifty-one telephone conversations (17 h), 244 written messages, and four interviews. Data were coded inductively, and themes constructed. The coding framework was subsequently applied to all post-study interviews (*n* = 20) to explore unmet needs.

**Results:**

Themes describing the roles of the dietitian were as follows: regular collaborative problem-solving to encourage empowerment, a reassuring care navigator including anticipatory guidance, and rapport building via psychosocial support. Psychosocial support included provision of empathy, reliable care provision, and delivery of positive perspective. Despite intensive counselling from the dietitian, nutrition impact symptom management was a core unmet need as it required intervention beyond the scope of practice for the dietitian.

**Conclusion:**

Delivery of nutrition care via the telephone or an asynchronous mobile application to people with newly diagnosed UGI cancer required the dietitian to adopt a range of roles to influence nutritional intake: they empower people, act as care navigators, and provide psychosocial support. Limitations in dietitians’ scope of practice identified unmet patient’s needs in nutrition impact symptom management, which requires medication management.

**Trial registration:**

27th January 2017 Australian and New Zealand Clinical Trial Registry (ACTRN12617000152325).

## Introduction

Patients with upper gastrointestinal (UGI) cancer (oesophagus, gastric, and pancreas) are vulnerable to malnutrition, with 48–90% diagnosed with malnutrition [1–3]. Symptoms of the cancer and its treatment are barriers to usual eating patterns that contribute to unintentional weight loss [4]. These symptoms include nausea, fatigue, dysphagia, oesophageal obstructions, early satiety, anxiety, depression, and anorexia [4]. Malnutrition impacts adversely on patients, with increased hospitalisation duration, reduced treatment efficacy, complications, and poorer survival [2, 5, 6]. Despite the importance of nutrition, these patients do not usually see a dietitian unless they are referred via the result of malnutrition screening, or directly from a nurse or doctor [3]. Evidence suggests, however, that 45% of dietetic referrals for patients with cancer should be provided earlier [7].

Limited dietetic resourcing and funding is a barrier to providing patients with cancer regular dietetic support, and therefore resource effective solutions are required [2, 8]. The Telephone or Electronic Nutrition care Delivery (TEND) study was a randomised controlled trial (RCT) that sought to overcome these barriers by testing non-traditional delivery modes of nutrition care including the telephone and a mobile application, to enable intensive, frequent dietetic intervention close to the time of diagnosis [9]. The dietetic intervention was provided external to the participant’s usual care team, and therefore the research dietitian was not part of a multidisciplinary team.

Identifying what people may need from a dietitian is important for designing best practice care [10]. Qualitative data describing people’s experience of care can enhance understanding of how an intervention sits within its context [10]. People with cancer are situated in a life-changing experience where diverse support needs are indicated [11]. The TEND study commenced before the rapid adoption of telehealth during the COVID-19 pandemic [12] and is a useful setting in which to explore the roles of a dietitian in delivering care via these delivery modes. Traditionally, dietitian-patient communications have been face-to-face; however, telephone and mHealth delivery remove traditional cues such as body language which may affect rapport and engagement between the dietitian and patient. Evidence is lacking, however, to explore the implications of these novel modes of delivery on the roles of the dietitian. Addressing this gap in evidence is important for delivering effective nutrition care to those undergoing treatment for cancer. The aim of the present study is to explore the patient-dietitian experience of an 18-week nutrition intervention (the TEND study) delivered using the telephone and a mobile application to people newly diagnosed with UGI cancer to elucidate the roles of the dietitian in this context.

## Materials and methods

This analysis is set within the TEND study, of which the RCT protocol has been outlined previously [13]. In brief, The TEND study was a three-arm RCT exploring the impact of delivering an 18-week intensive nutrition intervention to patients newly diagnosed with UGI cancer (oesophageal, gastric, and pancreatic cancer). The intervention was delivered by an experienced oncology dietitian, who provided personalised nutrition counselling and goal setting, with behaviour change strategies as informed by the Behaviour Change Technique Taxonomy (v1) [13–15]. A detailed representation of the dietetic intervention has been published [14]. Participants were allocated to receive the intervention using either the telephone or a mobile application, myPace. The initial nutrition assessment was conducted on the telephone. For proceeding weeks, the patient and dietitian communicated using the allocated mode of delivery. At least fortnightly communication was expected for intervention fidelity. A sub-sample of participants completed a post-study telephone interview (semi-structured with a pilot-tested question guide). The post-study interview questions have been described previously [16]. Briefly, the interviews explored patient acceptability of the novel modes of intervention delivery.

### Methodology

To examine the roles of the dietitian within the boundaries of the intervention, this study adopted Yin’s qualitative case study approach, from a post-positivist standpoint [17]. Although an uncommon choice, qualitative research methods may be used to align with a post-positivist viewpoint through researcher reflexivity and consideration of various explanations of the data [18, 19]. Case study analysis is an in-depth exploration of a phenomenon within a real-world situation [17, 20]. The ‘case’ for this study is the intervention, an approach that has been used previously [21–23]. This methodology was selected to allow an in-depth analysis of the patient-dietitian experience of the intervention whereby its boundaries included the dietitian, the patient, the frequency and duration of communication, and the tool for communication (i.e. a telephone or a mobile application). In this study, it was theorised that the dietitian’s key roles would be to provide the patient with psychosocial support, nutrition impact symptom management, nutrition optimisation, and pharmacological support. The validity of this theory was explored through analysing in-depth the patient-dietitian experiences within the intervention, as well as the post-study perspectives of patients, to determine the roles of the dietitian required during intervention delivery. The intervention delivery is explored in a real-world, uncontrolled context.

### Reflexivity

The research team included a student dietitian (S.T.), the study dietitian (K.F.), an experienced dietitian and qualitative researcher (T.C.), and a nutrition scientist with a scientific inquiry background (C.E.H.). These unique perspectives challenged presumptions of the roles of the dietitian in the described patient experience. As an aspiring dietitian, the student researcher interpreted the data more positively; however, this was managed through conversations with other researchers.

### Case selection

In total, six cases (i.e. experiences) of the intervention were selected using maximum-variation sampling [24]. Four of the six cases analysed were selected by the study dietitian, as these were participants who engaged in regular reviews with the dietitian, which is potentially a source of selection bias; therefore, an additional two were independently selected by S.T. to maximise variation of the intervention experiences, including mode of telehealth delivery, cancer type, and gender.

### Data collection

Within-intervention phone calls, messages, emails, and post-intervention interviews were audio-recorded/stored. The conversations within the 18 weeks of the six intervention experiences (both written and audio-recorded) were the primary sources of data for the case study. Post-intervention interviews (transcribed) where available were used as a data source in the case analysis. All post-intervention interviews (*n* = 20) were available to further examine the theme related to unmet needs.

### Data analysis

Data files from the six cases were uploaded to NVivo release 1.4.1 (QSR International, Melbourne, Australia) where the researcher (S.T.) inductively coded within-intervention phone calls, myPace messages, emails, and post-intervention interviews for each intervention experience sequentially. Dialogues from both the dietitian and patient were coded. A subset of transcripts were presented to another author (T.C.) for duplicate coding. Yin’s general strategy ‘Relying on theoretical propositions’ was used [17]. These propositions were used to help guide the coding process and were based on the theory of patient empowerment, the Supportive Care Framework, and anticipatory guidance [11, 25–27]. Audio files were coded directly on NVivo. An annotation was made each time a section of data was coded to provide context to the section coded. After coding was completed for an intervention experience, Yin’s analytic technique ‘Explanation building’ was used [17]. This involved reflecting on the case of the intervention and looking back to the initial theoretical propositions to see if changes were indicated. After this, Yin’s general analytic strategy ‘Examining rival explanations’ provided a step to reflect on alternative views of the data [17]. Codes were examined to create groups and sub-groups leading to the identification of key themes. S.T. consulted with C.E.H. to discuss code interpretation and theme identification. During this analytic process, a coding framework was developed.

The coding framework was subsequently used to guide analysis of the post-intervention interviews (*n* = 20) to gain a deeper understanding of the unmet needs of participants relating to symptom management. This information was extracted and included in the paper by the study dietitian (K.F.).

## Results

Characteristics of the patients receiving each intervention experience are described in Table 1. In total, 51 telephone conversations (17 h), 244 written messages, and four interviews were analysed from six participants.

**Table 1:** Characteristics of six patients who received an 18-week nutrition intervention for upper gastrointestinal cancer. These patients’ experiences of the intervention were analysed as part of the present case study

Intervention delivery mode	Participant ID	Cancer type	Gender	Age (years)	Completed weeks^a^	Post-study interview (yes/no)
Mobile application	1	Oesophageal	Male	57	13	No^c^
	2^b^	Gastric	Female	70	9	Yes
	3	Gastric	Female	61	14	Yes
Telephone	4	Pancreatic	Male	88	15	No^c^
	5	Oesophageal	Male	56	18	Yes
	6	Pancreatic	Female	55	15	Yes

^a^A completed week was defined as any week in the intervention where both the patient and dietitian communicated with each other. Weeks where the dietitian and patient planned no intervention are not included

^b^Intervention completed using myPace (7 weeks) and email (4 weeks), whereby both email and myPace were used to communicate for 2 weeks

^c^Post-study interview not completed as interviews were delivered to a subset of the RCT participants, and all interviews had been completed before this participant completed their intervention

### Key roles of the dietitian

**Fig. 1:**
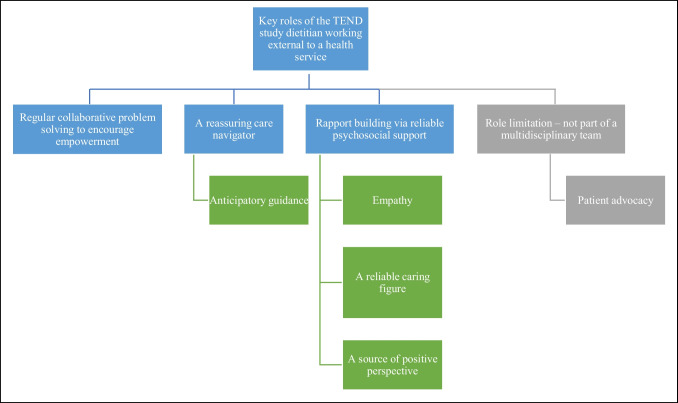
The key roles of the dietitian during an 18-week nutrition intervention delivered using the telephone and a mobile application, close to time of diagnosis for cancer of the upper gastrointestinal tract. The blue boxes represent the key roles of the dietitian, i.e. three key themes of this study. The green boxes represent any sub-themes of the key roles portrayed by the dietitian in the intervention. The grey boxes show the limitation of the role of the dietitian in the TEND study, the fourth key theme, and highlight that patient advocacy is impacted when working external to a multidisciplinary team

The themes describing key roles of the dietitian were (1) regular collaborative problem-solving to encourage empowerment, (2) reassuring care navigation (including anticipatory guidance), (3) rapport building via reliable psychosocial support, and (4) role limitations lead to unmet nutrition needs. These themes are detailed below and can be seen in Fig. 1.

#### Regular collaborative problem-solving to encourage empowerment

Patients experienced nutrition impact symptoms such as nausea, taste changes, obstructions, fatigue, and loss of appetite that impacted on eating. Symptoms experienced varied from patient-to-patient, and week-to-week, depending on factors such as cancer type, treatment modality, and cancer progression. These symptoms and the consequent loss of weight were a source of anxiety for patients. Patients were uncertain of how to manage eating and drinking with these symptoms and required the dietitian’s support.

…I have also lost my appetite and find it difficult to find a meal that I can eat and enjoy without feeling nauseous… I don’t know what to do. I have lost about 3 kilograms in weight since I last wrote. I will see the [usual care] dietitian at the trial centre on Monday and hope she will have an answer for me…. (Participant 2, myPace, week 10)

Dietary advice provided by the dietitian (verbally and in written education material) to achieve goals of weight stability in cancer was sometimes in conflict with dietary advice received prior to cancer diagnosis for other health issues. Having regular contact with the dietitian helped patients prioritise dietary instructions and removed the angst that came with trying to decipher conflicting advice leading to greater confidence to self-manage nutrition needs.

On many occasions, the patient was unnecessarily worried about their weight status, eating and drinking strategies, or food safety practices, and reassurance from the dietitian alleviated this concern. The prompt and regular delivery of reassurance by the dietitian was empowering for the patient. The dietitian also played an active role in encouraging self-confidence. This included illustrating to the patient when they were managing an issue appropriately and recognising patient achievements. Other forms of empowerment included encouraging social connection and patient participation in self-care. At study cessation, there was a sense that patients felt empowered in their own self-management capabilities.

I’ve learnt so much from interacting with [the dietitian] … and I think that’s the best part of it, that a patient has a chance to interact with a professional like that, and be able to help themselves. (Participant 2, myPace and email, post-study interview)

#### A reassuring care navigator

Cancer symptoms were worrying to the patient and the dietitian gave reassurance and prompted them to contact their medical practitioner to improve symptom management. This was a priority for the dietitian as poor symptom control impacts adversely on oral intake. Patients had concerns related to the use of some medications treating nutrition impact symptoms and the dietitian promoted understanding of these medications based on prescribing recommendations. When medication had been prescribed to a patient, the dietitian was able to support patients to apply the education information they received in hospital. For example, regular discussion with the dietitian was required to better understand how to dose pancreatic enzyme replacement therapy (PERT) with changes to oral intake.

[The dietitian] kind of set me – Set me right on how I should be taking it [PERT]… Um, things that I was unaware of, that I thought I was taking them properly and, you know, I wasn’t. (Participant 6, telephone, post-study interview)

Patients also discussed feedback received from other health professionals with the dietitian, indicating trust in her opinion.

… I’ve just had a call from [anonymous] in the Chemo Day Unit & apparently my pre-chemo blood test shows my current blood sugar levels are at 7.1. Normal should be between 4 & 6, so I have been referred to the Diabetes Unit & they will be in touch with me to arrange an appointment… Is this a normal process for people with a slightly high reading or…………….? (Participant 1, mobile application, week 16)

The dietitian was able to provide interpretation of this feedback and reassure them when they received positive news; i.e., the patient was unable to interpret the information in this way themselves. Additionally, the dietitian validated the patient of their right to play an active role in their treatment, i.e. to be informed, to make choices that aligned with their values and needs, and to speak up to healthcare providers where required, promoting patient-centred care.

#### Anticipatory guidance to minimise the consequences of nutrition impact symptoms

Because the intervention allowed for regular consultations, the dietitian was able to provide patients with information about what to expect along their cancer treatment. This included preparation for post-operative interventions, e.g. enteral feeding and fasting-related weight loss. Preoperative eating recommendations, for example carbohydrate loading, were also provided. On the other hand, when patients began stabilising their weight and hoping to gain, the dietitian realigned their expectations, with weight maintenance being the priority. From the patient perspective, this aspect of preparation was acknowledged.

…I think I got looked after; I really do. Preparation-wise. Yep, umm, and my weight sort of went up before surgery, so that was good. As soon as the chemo stopped, I started to put some weight on. (Participant 3, mobile application, post-study interview)

Preparatory advice relating to nutrition impact symptoms and their management was also provided by the dietitian. This included preparing patients for symptoms they may experience with chemotherapy/radiotherapy or after surgery and explaining the likely manifestation of different symptoms with treatment progression. The dietitian also prepared patients for any notable side effects they may experience with a new medication.

#### Rapport building via reliable psychosocial support

Evidence of rapport between the dietitian and the patient was apparent in both modes of the intervention. This was shown through moments of shared humour, and when a patient disclosed something personal or offered well wishes to the dietitian. Rapport building was enabled by the positive perspective, empathy, and reliability of the dietitian.

Patients felt comfortable disclosing their personal feelings and events to the dietitian, such as admitting when they were experiencing low mood or were finding something challenging or frustrating. Soon after their diagnosis, one patient described experiencing ‘*the couldn’t be bothereds*’, i.e. not feeling energised to do anything, and the dietitian positioned the patient to feel comfortable expressing personal feelings through empathising with their experiences and emotions.

The dietitian promoted positive affect. In the context of negative experiences that patients faced with eating and drinking, a role of the dietitian was to encourage patients to find enjoyment in eating. When patients disclosed exciting upcoming personal events, achievements, or other good news, the dietitian expressed excitement. Social support that patients received from family or friends was also highlighted and celebrated by the dietitian.

From the patient perspective, the dietitian was valued as someone to talk to who was a professional and not family or friends. Enjoyment was found in these conversations and gratitude was expressed to the dietitian for ‘*Being there*’*.*

…And thank you very much for what you’re doing …it’s good encouragement and that… Yes, it helps me think I’m getting somewhere. (Participant 4, telephone, week 8)

For the mobile application participants, comfort was found knowing that messages would be responded to, suggesting that the dietitian was valued as a reliable source of support.

…It was so good, because, you know, she didn’t really know me - I mean, you just develop a friendship, there’s no doubt about it, the kindness in her voice and that, but um, you know, once it’s finished, it’s finished. And um, yeah, so you can open up. (Participant 3, mobile application, post-study interview)

#### Role limitations lead to unmet nutrition needs

The case analysis revealed a myriad of unmet needs described by participants throughout the 18-week intervention period. To explore this further, an analysis of the post-intervention interviews (*n* = 20) was undertaken. Symptom managements, particularly those affecting the patient’s ability to eat and drink adequately, were those of primary concern for the dietitian to address. Many of these debilitating nutrition impact symptoms occurred at different intervals and were of differing intensity throughout the intervention period, and many individuals had multiple different symptoms competing for priority management during this time.

Not being able to eat. Physically, not mentally. I mean, I used to force food down my throat to the point where I was, you know, dry retching and I couldn’t eat because I was losing so much weight. But eating, that was the most challenging. (Participant 7, post-study interview)

The centralised nature of the dietetic intervention meant that many of the physical symptoms of anorexia, dry retching, nausea, and diarrhoea were unable to be managed effectively without the collaboration of a multidisciplinary team where the dietitian could advocate for medications to be reviewed. Often, where medication was prescribed to manage these symptoms, its dosing and mechanism for action were not explained or understood by the patients.

“I’m on four Creon [PERT]. Is that right?” She [the dietitian] said, “No, no, back to two.” Instantly the bowels stopped feeling bloated and gassy and all that jazz, it was much better for me. (Participant 8, post-study interview)

The dietitian provided recommendations for participants to contact their general practitioner, oncologist, or surgeon to prescribe medications; however, this required patient advocacy which was often delayed. Delays were caused by appointment time delays, a competing schedule of chemotherapy and/or other appointments, and physical access such as transport.

## Discussion

This study explored the patient-dietitian experience during an 18-week nutrition intervention delivered using the telephone or a mobile application to people newly diagnosed with UGI cancer to elucidate the roles of the dietitian during the intervention. This study identifies important components of delivering supportive care via different modes for those undergoing treatment for UGI cancer. The roles of the dietitian were characterised by regular collaborative problem-solving to encourage empowerment, a reassuring care navigator (including anticipatory guidance), and rapport building via reliable psychosocial support. This analysis also revealed that role limitations led to unmet needs as the dietitian was constrained by poorly managed cancer symptoms that negatively impacted oral intake and subsequently weight stability.

This study demonstrated that rapport can be built within the patient-dietitian relationship without face-to-face communication. It is well established that psychosocial support, for example empathy, compassion, positivity, reassurance, and care, is valued by patients in the dietitian-patient relationship [28, 29], and this study shows it can be achieved over the telephone and via asynchronous modes of communication. In addition to psychosocial support, previous research indicates that patients value having a point of contact in their cancer journey [30]. In the present study, it appeared that the dietitian fulfilled this role of a central point of contact for the patients, making parallels with the role of a patient care navigator. Patient care navigators can take the form of a nurse, social worker, case manager, and laypersons [31]. Broadly, their role is to facilitate patients and their family through their cancer experience to increase access to required resources and reduce anxiety. Their role may include problem-solving, facilitating symptom management, liaising with the multidisciplinary team, and making referrals [31]. Likewise, the dietitian in this study provided anticipatory guidance, problem-solving for symptom management, and guidance when contact with another health professional was indicated. Both health professionals and patients recognise the value of the oncology dietitian’s ability to prepare patients for common challenges that occur throughout the cancer journey [16, 28, 32, 33]. Evidence suggests that patient navigation services benefit patients through addressing worries and reducing incidence of hospital admissions and emergency department attendance [34–36]. This study proposes that the care navigator role does not need to be limited to nursing or social work, and that, with training and education, could be shared by the multidisciplinary team. This would not only provide more human resources for this role, but it would also act to benefit the health professional through broadening their knowledge and connection to the patient experience through continuity of care, ultimately promoting patient-centred care. Furthermore, use of telephones and electronic devices in the delivery of care navigation could be an effective and time-efficient way to deliver care navigation, enabling flexibility and ease of access between the patient and their navigator.

The dietitian’s role as a care navigator and multidisciplinary team member was limited by the scope of the RCT as the dietitian could only recommend to the patient when and where to seek help. It was not within the scope of the intervention for the dietitian to actively contact patients’ individual healthcare team to advocate, discuss, and collaborate to optimise symptom and medication management, distress, and treatment progression. This highlights the importance of multidisciplinary teams being critical in cancer management and the need for the dietitian to be part of the team. Improving the safety and quality of health service provision to ensure that it is provided by the right person, at the right time, in the right place, and for the right cost is essential [37, 38]. The dietitian can play an integral role in extended or advanced practice roles to address the high prevalence of malnutrition and high levels of unmet needs particularly relating to nutrition impact symptoms, and act as a care navigator to guide individuals and their families/carers on their cancer journey.

The case study design is a strength of this work; many studies have short-term interventions and provide little insight into how the intervention works. The use of within-intervention conversations was also a strength of this study as this data is not limited by memory or response bias. This detailed analysis reveals the roles of the dietitian needed to maximise oral intake during cancer treatment; furthermore, it reveals that sub-optimal cancer symptom management remains a significant barrier to achieving optimal oral intake, even when under the intensive care of a dietitian. A limitation is that this was a post hoc analysis, precluding direct questions of the patient and dietitian perspective of the dietitian’s role.

## Conclusion

This study explored the patient-dietitian experience of an 18-week nutrition intervention delivered non-face-to-face using the telephone or a mobile application to patients with newly diagnosed UGI cancer. Regular collaborative problem-solving to encourage empowerment, reassuring care navigation (including provision of anticipatory guidance), and rapport building via reliable psychosocial support were key roles of the dietitian. It is evident that patients with UGI cancer are faced with nutrition impact symptoms that limit the capacity of the dietitian to support patients in isolation of the multidisciplinary team. Further, research is recommended to examine an advanced care role for dietitians in the management of nutrition impact symptoms.

## References

[1] Loeliger J, Kiss N (2015) Phase II malnutrition in Victorian Cancer services: summary report. Dept of Health and Human Services, State Government of Victoria, Melbourne

[2] Marshall K, Loeliger J (2013) Investigating practices relating to malnutrition in Victorian cancer services: summary report 2012. Dept of Health, State Government of Victoria. https://www2.health.vic.gov.au/about/health-strategies/cancer-care/cancer-projects/investigating-practices-relating-to-malnutrition-in-victorian-cancer-services. Accessed 4 June 2021

[3] Silvers MA, Savva J, Huggins CE, Truby H, Haines T (2014). Potential benefits of early nutritional intervention in adults with upper gastrointestinal cancer: a pilot randomised trial. Support Care Cancer.

[4] Cancer Council Victoria and Department of Health Victoria (2021) Optimal care pathway for people with oesophagogastric cancer. https://www.cancer.org.au/assets/pdf/oesophagogastric-cancer-optimal-cancer-care-pathway. Accessed 4 June 2021

[5] Andreyev HJ, Norman AR, Oates J, Cunningham D (1998). Why do patients with weight loss have a worse outcome when undergoing chemotherapy for gastrointestinal malignancies?. Eur J Cancer.

[6] Van Cutsem E, Arends J (2005). The causes and consequences of cancer-associated malnutrition. Eur J Oncol Nurs.

[7] Lorton CM, Griffin O, Higgins K, Roulston F, Stewart G, Gough N, Barnes E, Aktas A, Walsh TD (2020). Late referral of cancer patients with malnutrition to dietitians: a prospective study of clinical practice. Support Care Cancer.

[8] Findlay M, Rankin NM, Shaw T, White K, Boyer M, Milross C, De Abreu LR, Brown C, Collett G, Beale P, Bauer JD (2020). Best evidence to best practice: implementing an innovative model of nutrition care for patients with head and neck cancer improves outcomes. Nutrients.

[9] Huggins CE, Hanna L, Furness K, Silvers MA, Savva J, Frawley H, Croagh D, Cashin P, Low L, Bauer J, Truby H, Haines TP (2022). Effect of early and intensive telephone or electronic nutrition counselling delivered to people with upper gastrointestinal cancer on quality of life: a three-arm randomised controlled trial. Nutrients.

[10] Rand L, Dunn M, Slade I, Upadhyaya S, Sheehan M (2019). Understanding and using patient experiences as evidence in healthcare priority setting. Cost Eff Resour Alloc.

[11] Fitch MI (2008). Supportive care framework. Can Oncol Nurs J.

[12] Rozga M, Handu D, Kelley K, Jimenez EY, Martin H, Schofield M, Steiber A (2021). Telehealth during the COVID-19 pandemic: a cross-sectional survey of registered dietitian nutritionists. J Acad Nutr Diet.

[13] Hanna L, Huggins CE, Furness K, Silvers MA, Savva J, Frawley H, Croagh D, Cashin P, Low L, Bauer J, Truby H, Haines T (2018). Effect of early and intensive nutrition care, delivered via telephone or mobile application, on quality of life in people with upper gastrointestinal cancer: study protocol of a randomised controlled trial. BMC Cancer.

[14] Furness K, Huggins CE, Hanna L, Silvers MA, Cashin P, Low L, Croagh D, Haines TP (2018). A process and mechanism of action evaluation of the effect of early and intensive nutrition care, delivered via telephone or mobile application, on quality of life in people with upper gastrointestinal cancer: a study protocol. BMC Cancer.

[15] Michie S, Richardson M, Johnston M, Abraham C, Francis J, Hardeman W, Eccles MP, Cane J, Wood CE (2013). The behavior change technique taxonomy (v1) of 93 hierarchically clustered techniques: building an international consensus for the reporting of behavior change interventions. Ann Behav Med.

[16] Furness K, Huggins CE, Truby H, Croagh D, Haines TP (2021). Attitudes of Australian patients undergoing treatment for upper gastrointestinal cancers to different models of nutrition care delivery: qualitative investigation. JMIR Form Res.

[17] Yin RK (2009). Case study research: design and methods.

[18] Grant BM, Giddings LS (2002). Making sense of methodologies: a paradigm framework for the novice researcher. Contemp Nurse.

[19] Monrouxe LV, Rees CE (2020). When I say … quantification in qualitative research. Med Educ.

[20] Crowe S, Cresswell K, Robertson A, Huby G, Avery A, Sheikh A (2011). The case study approach. BMC Med Res Methodol.

[21] Lesage-Moussavou-Nzamba M, Houle J, Trudeau F (2020) Participants’ perspectives of a primary exercise-based prevention program for cardiac patients: a prepost intervention qualitative case study. Rehabil Res Pract. 10.1155/2020/6215428 10.1155/2020/6215428 PMC7180414 32351738

[22] Rotteau L, Albert M, Bhattacharyya O, Berta W, Webster F (2021). Understanding decisions to scale up: a qualitative case study of three health service intervention evaluations. J Health Serv Res Policy.

[23] Moll SE (2014). The web of silence: a qualitative case study of early intervention and support for healthcare workers with mental ill-health. BMC Public Health.

[24] Given LM (2008). The SAGE Encyclopedia of Qualitative Research Methods.

[25] Alpay LL, Henkemans OB, Otten W, Rövekamp TAJM, Dumay ACM (2010). E-health applications and services for patient empowerment: directions for best practices in The Netherlands. Telemed J E Health.

[26] Aujoulat I, d’Hoore W, Deccache A (2007). Patient empowerment in theory and practice: polysemy or cacophony?. Patient Educ Couns.

[27] Johnson EL, Frias JP, Trujillo JM (2018). Anticipatory guidance in type 2 diabetes to improve disease management next steps after basal insulin. Postgrad Med.

[28] McCarter K, Baker AL, Britton B, Halpin SA, Beck A, Carter G, Wratten C, Bauer J, Wolfenden L, Burchell K, Forbes E (2018). Head and neck cancer patient experience of a new dietitian-delivered health behaviour intervention: ‘you know you have to eat to survive’. Support Care Cancer.

[29] Sladdin I, Chaboyer W, Ball L (2018). Patients’ perceptions and experiences of patient-centred care in dietetic consultations. J Hum Nutr Diet.

[30] Easley J, Miedema B, Carroll JC, O’Brien MA, Manca DP, Grunfeld E (2016) Patients’ experiences with continuity of cancer care in Canada: results from the CanIMPACT study. Can Fam Physician 62(10):821–827 PMC5063773 27737982

[31] McMullen L (2013). Oncology nurse navigators and the continuum of cancer care. Semin Oncol Nurs.

[32] Furness K, Huggins C, Croagh D, Haines T (2021). Exploring the attitudes of health professionals providing care to patients undergoing treatment for upper gastrointestinal cancers to different models of nutrition care delivery: a qualitative investigation. Nutrients.

[33] Hazzard E, Walton K, McMahon A-T, Milosavljevic M, Tapsell LC (2021). Healthcare professionals’ perspectives on the role of dietitians within multidisciplinary head and neck cancer teams: a qualitative multi-site study. Nutr Diet.

[34] Kline RM, Rocque GB, Rohan EA, Blackley KA, Cantril CA, Pratt-Chapman ML, Burris HA, Shulman LN (2019). Patient navigation in cancer: the business case to support clinical needs. J Oncol Pract.

[35] Rocque GB, Partridge EE, Pisu M, Martin MY, Demark-Wahnefried W, Acemgil A, Kenzik K, Kvale EA, Meneses K, Li X, Li Y, Halilova KI, Jackson BE, Chambless C, Lisovicz N, Fouad M, Taylor RA (2016). The Patient Care Connect Program: transforming health care through lay navigation. J Oncol Pract.

[36] Rocque GB, Pisu M, Jackson BE, Kvale EA, Demark-Wahnefried W, Martin MY, Meneses K, Li Y, Taylor RA, Acemgil A, Williams CP, Lisovicz N, Fouad M, Kenzik KM, Partridge EE, Patient Care Connect Group (2017). Resource use and Medicare costs during lay navigation for geriatric patients with cancer. JAMA Oncol..

[37] Australian Commission on Safety and Quality in Healthcare, About Us. https://www.safetyandquality.gov.au/about-us. Accessed 15 Aug 2022

[38] Beesley VL, Janda M, Goldstein D, Gooden H, Merrett ND, O’Connell DL, Rowlands IJ, Wyld D, Neale RE (2016). A tsunami of unmet needs: pancreatic and ampullary cancer patients’ supportive care needs and use of community and allied health services. Psychooncology.

